# Accuracy Assessment of Customized Titanium Plates Compared to 3D-Printed Splints in Le Fort I Osteotomy: A Randomized Clinical Trial Evaluating Clinical and Radiographic Outcomes

**DOI:** 10.7759/cureus.84151

**Published:** 2025-05-15

**Authors:** Abdelkareem M Abdelhamid, Atef M Hassan, Wael A El-Mohandes

**Affiliations:** 1 Oral and Maxillofacial Surgery, Faculty of Dental Medicine, Al-Azhar University, Cairo, EGY

**Keywords:** 3d-printed splints, customized titanium plate, le fort 1 osteotomy, orthognathic surgeries, surgical accuracy

## Abstract

Aim: This study aimed to evaluate the accuracy of a personalized orthognathic surgical guide (POSG) system using customized titanium fixation plates versus 3D-printed splints for maxillary repositioning in Le Fort I osteotomy.

Trial design: A randomized clinical trial with parallel-arm design and 1:1 allocation ratio with a superiority framework.

Methods: Twelve patients requiring Le Fort I osteotomy were randomly assigned to two groups based on the surgical transfer technique: a customized titanium plate group (study group) and a 3D-printed splint group (control group). Virtual surgical planning was performed using Mimics Medical 19.0 (Materialise, Leuven, Belgium) and 3-Matic Medical 14.0 (Materialise, Leuven, Belgium) software to generate stereolithography (STL) files for cutting guides and fixation plates. Preoperative and postoperative CT scans were aligned using surface-based, landmark-free registration. Deviations from the surgical plan and postoperative CT scan were measured at selected anatomical landmarks across three reference planes: the Frankfort horizontal plane (FHP), midsagittal plane (MSP), and coronal plane (CP). Primary outcomes included the accuracy of surgical transfer. Secondary outcomes included operative time, fabrication accuracy, hardware complications, and postoperative infection.

Results: There were no statistically significant differences between the virtual plan and postoperative outcomes for MSP and FHP measurements in either group (p > 0.05). However, a statistically significant difference was observed in CP measurements, with the control group showing a greater mean deviation (1.54 ± 0.28 mm) than the study group (1.08 ± 0.25 mm; p = 0.013). The mean operative time was significantly shorter in the customized plate group (126.67 ± 11.69 minutes) than in the 3D splint group (178.33 ± 15.38 minutes; p < 0.001). All patients achieved stable postoperative occlusion. One patient in the customized plate group experienced transient upper lip numbness, which resolved within two months. No plate loosening, infection, or soft tissue complications were reported.

Conclusion: Both customized titanium plates and 3D-printed splints accurately transferred virtual surgical plans for Le Fort I osteotomy. However, the personalized plate system showed greater precision in anteroposterior maxillary positioning. Additionally, it reduced surgical time and eliminated the need for intraoperative splint use. Further studies with larger sample sizes and standardized protocols are recommended to validate these findings and guide future clinical applications.

## Introduction

Dentofacial deformity (DFD) is a multifactorial condition affecting the maxilla and mandible's size, shape, and spatial relationship. Individuals with dentofacial deformities frequently experience challenges related to chewing, speaking, and breathing, in addition to psychological and aesthetic concerns that may adversely affect their overall [1].

Orthognathic surgery is a well-established intervention for correcting moderate to severe dentofacial anomalies. Its primary goals are to restore normal occlusion and facial proportions while improving function and long-term skeletal stability [2]. Over the years, both surgical techniques and preoperative planning methods have evolved significantly to enhance surgical precision and outcomes [3].

Traditional methods of maxillary repositioning, such as model surgery and hand-fabricated interocclusal splints, have limitations. These include potential inaccuracies in splint fabrication, difficulties in vertical control, and variations in intraoperative condylar positioning, all of which can compromise the surgical outcome [4-6]. Vertical positioning is particularly challenging, as it often relies on intraoral or extraoral landmarks, such as nasion screws or glabella pins [7-9].

To overcome such challenges, three-dimensional virtual surgical planning (3D-VSP) has emerged as a valuable tool, offering improved accuracy in simulating and forecasting surgical results [6]. Transfer of these virtual plans to the operative field typically relies on one of two approaches: the use of intermediate splints (splint-based) or the application of customized surgical navigation and fixation plates (splintless) [10,11].

Although 3D-printed splints derived from virtual plans have shown improved accuracy compared to traditional methods, they are still prone to seating errors and challenges in maintaining accurate vertical positioning during surgery. In contrast, Virtual planning allows for the fabrication of patient-specific titanium fixation plates, which, when used alongside cutting guides, enable precise bone positioning, reduce surgical duration, and eliminate the dependence on occlusal splints or intermaxillary fixation, with a lower risk of condylar sagging [12,13].

This study aims to evaluate and compare the accuracy of a personalized orthognathic surgical guide (POSG) system (consisting of cutting guides and custom 3D-printed titanium plates) with that of a conventional 3D-printed splint system for Le Fort I maxillary repositioning in orthognathic surgery.

## Materials and methods

Trial design

Randomized, parallel-arm clinical trial with a 1:1 allocation ratio and a superiority framework. The individual participant was the unit of randomization and analysis. The trial protocol was registered prospectively in the clinicaltrials.gov registry with the trial identification number NCT06933628. The study was reported according to the updated CONSORT (Consolidated Standards of Reporting Trials) guidelines for reporting randomized clinical trials [14].

Trial setting

The trial was conducted in a single center (Faculty of Dental Medicine Hospital, Al-Azhar University), where participants were recruited from the outpatient clinic of the Oral and Maxillofacial Surgery Department, Faculty of Dental Medicine, Al-Azhar University, Cairo, Egypt. Ethical approval was obtained from the Research Ethics Committee of the Faculty of Dental Medicine, Al-Azhar University (Ethical Code: 887/1849, dated November 22, 2022). All the eligible participants signed a written informed consent form before enrolling in the study and received a detailed explanation of the study objectives and procedures. The study duration was from January 10, 2023, to April 1, 2025.

Eligibility criteria

Participants referred to the outpatient clinic of the Oral and Maxillofacial Surgery department and diagnosed with dentofacial deformities were considered for enrollment in the study. All the eligible participants were diagnosed with dentofacial deformities that required surgical correction with Le Fort I osteotomy. Participants were healthy (American Society of Anesthesiologists (ASA) I or II classification) and aged 18 to 30. Participants were excluded from the study if they had a history of cleft palate or other craniofacial anomalies, required segmental Le Fort I osteotomy, were pregnant or suspected of being pregnant, or had a previous history of orthognathic surgery.

Interventions and comparators

**Figure 1:**
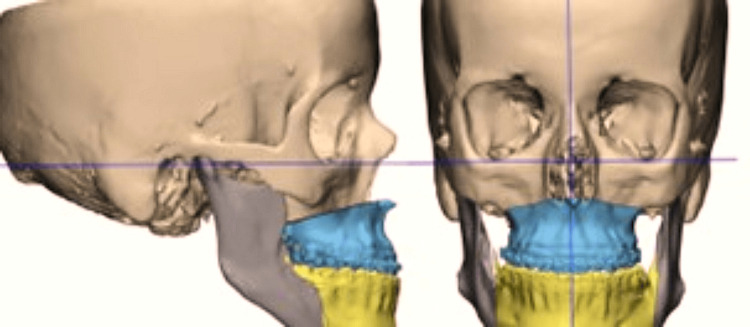
Virtual planning of the osteotomy after 3D reconstruction of a full skull CT scan.

**Figure 2:**
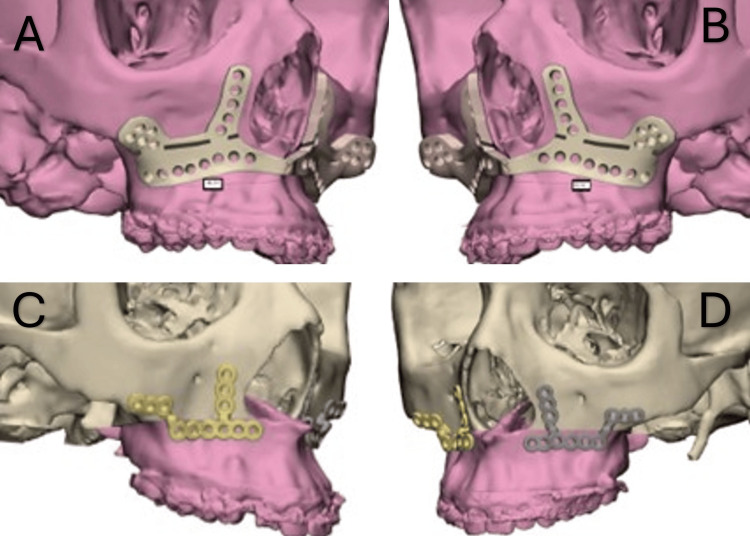
Virtual planning and design of the cutting guides and the customized titanium plates. (A & B) Virtual design of the cutting guides; (C & D) virtual design of the right and left customized titanium plates.

Twelve participants were enrolled and randomized to the study groups. All participants underwent a standardized preoperative evaluation, which included a thorough clinical examination, dental and medical history review, and radiographic assessment. Diagnostic imaging included lateral cephalometric radiographs to confirm the skeletal diagnosis, panoramic radiographs to assess dentition, root anatomy, intraosseous lesions, and impacted teeth, and complete skull computed tomography (CT) scans for three-dimensional evaluation. The Digital Imaging and Communications in Medicine (DICOM) files obtained from the CT scans were imported into Mimics Medical 19.0 software (Materialise, Leuven, Belgium) to create 3D virtual models for surgical planning. These models were then exported as stereolithography (STL) files and processed using 3-Matic Medical 14.0 (Materialise, Leuven, Belgium). Customized cutting guides, titanium fixation plates, and 3D-printed surgical splints were designed using the software’s advanced computer-aided design (CAD) tools based on the individualized virtual surgical plans (Figures 1, 2).

Surgical technique and preparation

**Figure 3:**
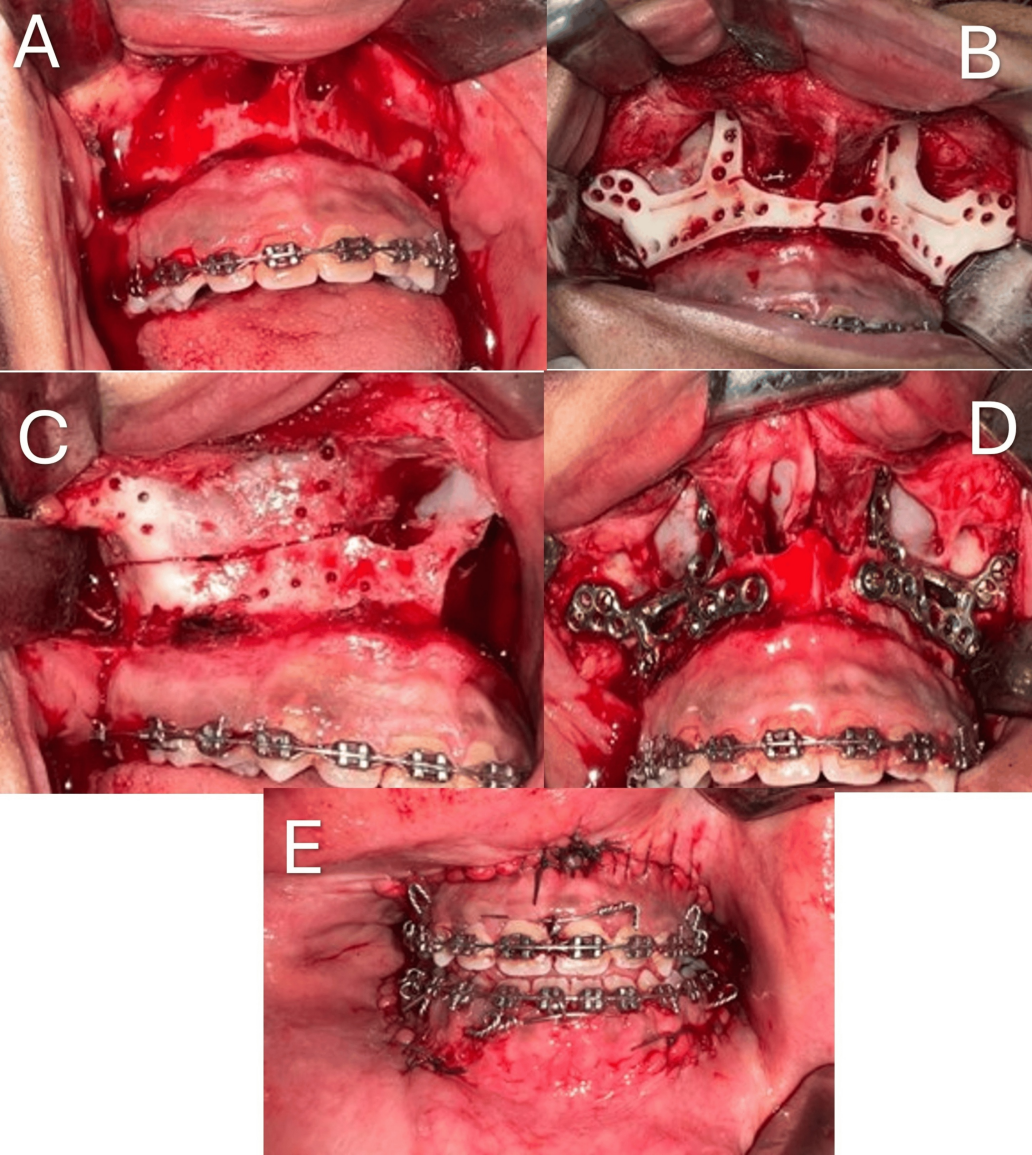
Surgical steps for the Le Fort I osteotomy. (A) Mucoperiosteal flap incised and elevated; (B) custom-made cutting guide in place; (C) line of osteotomy and screw holes; (D) customized titanium plate fixation; (E) Suturing of the flap postoperative occlusion.

All patients were admitted to the hospital and underwent standard preoperative laboratory investigations. Surgical procedures were performed under hypotensive general anesthesia to minimize intraoperative bleeding and improve surgical field visibility. All patients underwent Le Fort I osteotomy in combination with bilateral sagittal split osteotomy (BSSO) to correct dentofacial deformities and achieve optimal occlusion and facial harmony (Figure 3).

For the customized titanium plate group (study group), an intraoral vestibular incision was made between the upper first molars, at least 5 mm above the mucogingival junction for the maxillary procedure. A full-thickness mucoperiosteal flap was elevated to expose the anterior maxilla. A two-piece customized cutting guide was positioned on the bone surface and fixed with four 2.0 mm screws to ensure stability during the marking of reference holes. A reciprocating saw marked the maxillary osteotomy line on the lateral wall. Following the removal of the guide, osteotomy cuts were completed with the same saw and refined using thin osteotomes at the posterior maxillary buttresses. The lateral nasal walls were sectioned using nasal chisels, and the nasal septum was separated with a septum osteotome. The pterygomaxillary junction was carefully separated using curved osteotomes, with intraoral finger palpation of the hamulus to confirm the integrity of the osteotomy.

Upon completion of the osteotomies, the maxilla was down-fractured using digital pressure and mobilized with Rowe’s disimpaction forceps. It was repositioned according to the preoperative virtual surgical plan and fixed using customized titanium plates secured with 2.0 mm mini-screws. A BSSO was performed for mandibular correction. Proper occlusion was verified, and final positioning was achieved based on the surgical plan. Buccal incisions in the maxilla and mandible were closed using 4-0 Vicryl (Ethicon, Somerville, NJ) in a continuous running suture to ensure watertight closure.

For the 3D-printed splint group, after completion of the Le Fort I osteotomy, the intermediate 3D-printed splint was adapted to the occlusal surfaces of the upper and lower teeth. Stainless steel wire (26-gauge) was used for intermaxillary fixation (IMF). The maxilla was fixed in its preplanned position using four titanium miniplates (2 mm), two at the zygomaticomaxillary buttresses and two at the piriform rims and monocortical screws (2 mm) on both sides. The plates were bent to fit passively on the bone surface. After removing the IMF, the mandibular BSSO was performed, and the final 3D-printed splint was adapted to the occlusal surfaces of the upper and lower teeth. The patient was placed in maxillomandibular fixation (MMF). The mandible was fixed in its final position using titanium miniplates and monocortical screws on both sides, and the IMF was removed.

Outcomes

The primary outcome of this study was the accuracy of transferring the virtual surgical plan to the actual surgical procedure. Secondary outcomes included operative time, the accuracy of fabricating surgical tools (cutting guides, customized plates, and 3D-printed splints), plate exposure or loosening, and postoperative infection.

The accuracy of the transfer system was assessed by measuring the discrepancies at five specific anatomical landmarks: the most infero-mesial point of the upper central incisor, the tips of the upper canines (right and left), and the mesiobuccal cusp tips of the upper first molars (right and left). These points were compared to three reference planes: the midsagittal plane (MSP), Frankfort horizontal plane (FHP), and the coronal plane (CP). The discrepancies between the preoperative virtual plan and the postoperative 3D model were measured in millimeters by aligning the preoperative and postoperative CT scans. The fabrication accuracy of the surgical guides and custom-made plates was evaluated by assessing their adaptation and fit to the preplanned positions during surgery. Specifically, the 3D-printed splint was checked for proper alignment with the occlusal surfaces of the maxillary and mandibular teeth. Operative time was measured from the initiation of the maxillary vestibular incision to the completion of plate fixation. The time was recorded in minutes. Additional complications, such as plate exposure, loosening, and postoperative infection, were monitored and documented if they occurred.

Sample size

The sample size calculation was based on a previous study [15]. Using G*Power statistical power analysis program (version 3.1.9.4) (Heinrich-Heine-Universität Düsseldorf, Düsseldorf, Germany) for sample size determination [16]. A sample size (n=12; subdivided to 6 in each group) was sufficient to detect a large effect size (t) ranging from 1.94 to 2.18, with an actual power (1-β error) of 0.8 (80%) and a significance level (α error) 0.05 (5%) for two-sided hypothesis test.

Randomization and blinding

The allocation sequence was generated using a computer-generated random sequence by Random.org with a 1:1 allocation ratio. Allocation was concealed using sequentially numbered opaque sealed envelopes containing folded papers with the type of intervention written on them. The allocation sequence generation and concealment were conducted by a researcher not involved in the surgical procedures or outcome assessments. This study employed a single-blinded design, where only the outcome assessor was blinded to the intervention. The operator and the patient were aware of the intervention due to the nature of the procedures.

Statistical methods

Recorded data were analyzed using IBM SPSS Statistics for Windows, Version 23 (IBM Corp., Armonk, NY). The quantitative data were presented as mean and standard deviation. The Shapiro-Wilk test was used to explore normality. An independent samples t-test was used to compare the means of the two groups. The level of statistical significance was set at 5%.

## Results

**Figure 4:**
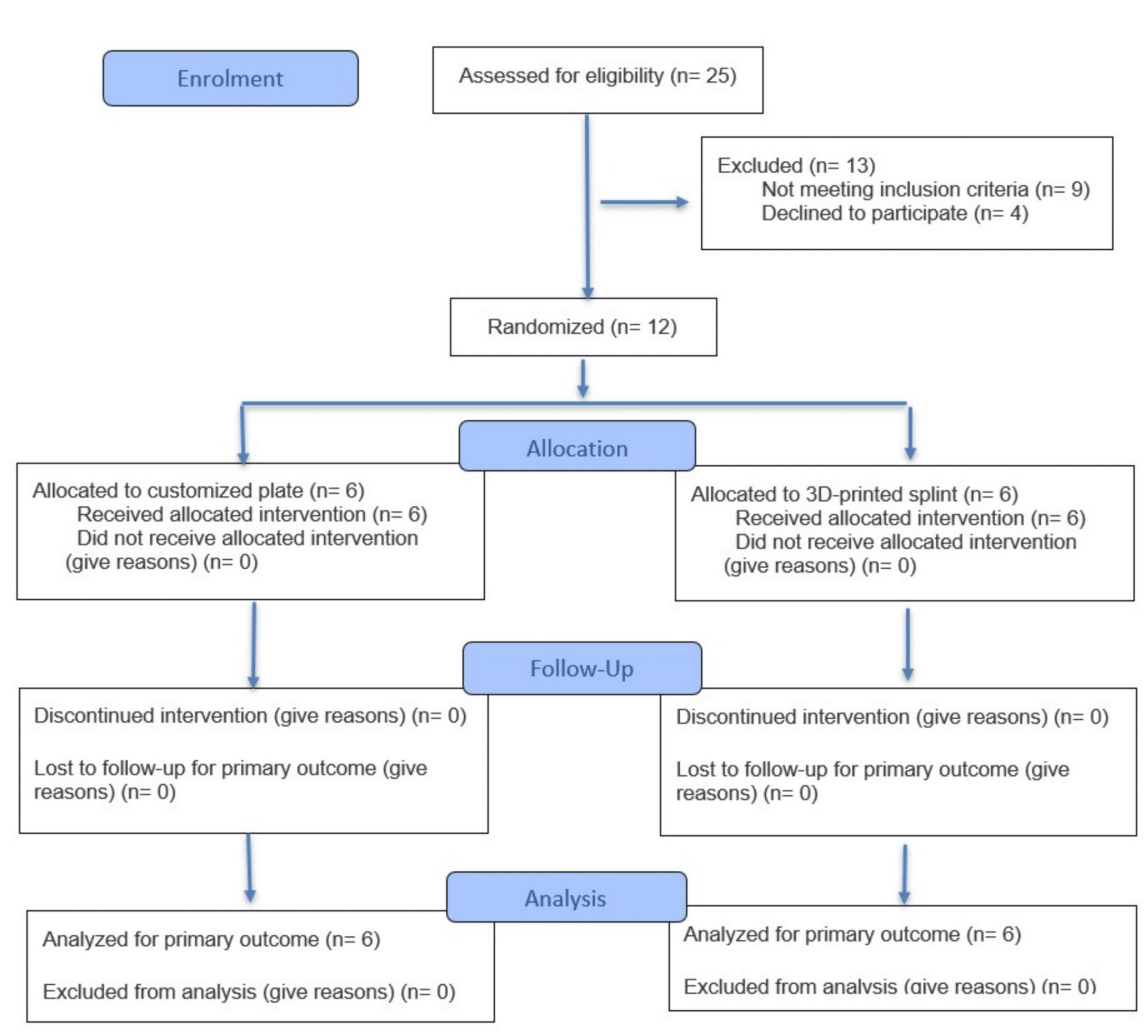
CONSORT flow diagram showing flow of the patients through the study. CONSORT: Consolidated Standards of Reporting Trials.

Twelve patients with dentofacial deformity were enrolled and randomized for Le Fort I osteotomy using different approaches. The VSP was translated to the patient using a customized titanium plate system and 3D-printed splints. All patients completed the follow-up successfully (Figure 4).

The data distribution was normal, as evident by a non-significant Shapiro-Wilk test (p-value > 0.05). There was no statistically significant discrepancy between the study and control groups regarding FHP and MSP (p-value > 0.05). However, the control group exhibited a significantly higher mean value of discrepancy of CP, with a p-value (p = 0.013), as shown in Table 1.

**Table 1:** Results of deviation of selected points between VSP and post-operative CT scan in three planes (MSP, FHP, and CP) in both groups. VSP: virtual surgical planning, SD: standard deviation, mm: millimeters, MSP: midsagittal plane, FHP: Frankfort horizontal plane, CP: coronal plane, *: statistically significant (independent t-test).

Planes	Custom-made plate group Mean ± SD	3D-printed splint group Mean ± SD	T value	P value
MSP	0.8 ± 0.26 mm	0.8 ± 0.2 mm	0.017	0.987
FHP	1.1 ± 0.23 mm	0.9 ± 0.16 mm	0.802	0.426
CP	1.08 ± 0.25 mm	1.54 ± 0.28 mm	3.002	0.013*

The 3D-printed splint group recorded a significantly longer mean procedure duration than the custom-made plate group (p = 0.0001), as shown in Table 2.

**Table 2:** Results of the duration of the surgical intervention in both groups in minutes. SD: standard deviation, *: statistically significant (independent t-test).

Variable	Custom-made plate group	3D-printed splint group	T value	P value
Duration in minutes Mean ± SD	126.67 ± 11.69 minutes	178.33 ± 15.38 minutes	-6.55	0.0001*

All the cases in this study from both groups exhibited minimal postoperative complications, with no instances of soft tissue dehiscence, infection, plate fractures, or screw loosening. One patient in the custom-made plate group reported a foul odor during breathing and was referred to an Ear, Nose, and Throat specialist for further assessment. All patients who underwent BSSO experienced numbness in the lower lip, which diminished over time and disappeared within three months for most cases, and six months for one case. Additionally, one patient reported slight numbness in the upper lip, which resolved within two months. Adaptation and fit of the fabricated surgical guides and custom-made plates showed good adaptation during surgery, except for one case in the custom-made plate group, which did not fit the bone surface in the upper right section.

## Discussion

This study included 12 patients with dentofacial deformities requiring Le Fort I osteotomy, randomly assigned to two groups. The conventional approach to maxillary repositioning has several limitations, including measurement inaccuracies, manual errors during splint fabrication, and procedural variability. These issues may result in significant maxillary positioning discrepancies, with malpositioning reported as high as 5 mm in some cases [17].

Several computer-aided design/computer-aided manufacturing (CAD/CAM)-generated surgical techniques have been developed to improve the accuracy of transferring virtual surgical plans into the operative field. These include 3D-printed occlusal wafers [18], pre-bent titanium plates [19], repositioning guides, surface templates [20-22], and, more recently, customized titanium plates [23]. Mazzoni et al. and Gander et al. were among the first to propose a fully customized system combining surgical guides and fixation plates [4,24]. Karanxha et al. described a similar design in a case report, while Suojanen et al. conducted a larger study involving 32 patients [15,25].

Our study assessed the accuracy of maxillary positioning by comparing planned and postoperative outcomes using distances from fixed anatomical landmarks to axial, coronal, and sagittal planes. Postoperative skulls were superimposed on virtual surgical plans using an initial N-point registration, followed by global registration to enhance precision. Although both voxel-based and surface-based registration methods are widely used, voxel-based registration is theoretically more accurate as it considers the entire 3D volume. However, it requires more advanced computational resources. Surface-based registration may be less comprehensive, but it remains a viable alternative. Almukhtar et al. reported no statistically significant difference in accuracy between these methods [26].

This study compared the accuracy of two surgical transfer methods: an intermediate 3D splint and a customized surgical guide with a fixation plate. Both approaches produced clinically acceptable discrepancies between planned and actual postoperative outcomes. However, the study group (customized plate group) showed significantly higher accuracy in the anteroposterior dimension, with a mean deviation of 1.08 (0.25) mm compared to 1.54 (0.28) mm in the control group. No significant differences were observed between MSP and FHP positioning groups.

These findings align with Kraeima et al., who found that patient-specific osteosynthesis (PSO) is particularly effective for planned anteroposterior translations exceeding 3.7 mm [27]. Similarly, Rückschloß et al. reported that PSO offers greater overall accuracy, especially in the anterior-posterior direction [28]. Furthermore, this study showed that the mean operative time was significantly reduced in the customized plate group (126.67 ± 11.69 minutes) compared to the control group (178.33 ± 15.38 minutes). The integration of CAD/CAM customized titanium plates eliminated the need for nasion reference screws, manual plate bending, and occlusal splint wiring, thus streamlining the procedure. Importantly, no differences were noted between groups regarding complications such as infections, soft tissue-related issues, or the need for plate removal.

The surgical guides, custom-made plates, and 3D-printed splints were evaluated for fabrication accuracy by assessing their adaptation and fit to the preplanned positions. The 3D-printed splint was checked explicitly for proper alignment with the occlusal surfaces of the maxillary and mandibular teeth. All appliances, surgical guides, custom-made plates, and 3D-printed splints demonstrated accurate fitting. However, exceptions were noted in one upper right customized plate did not fit the bone this hole left without screw and there was rocking of the intermediate and final splints on the occlusal surface was observed. This splint was adjusted to fit on the occlusal surface of the teeth.

This study's results suggest that using CAD/CAM 3D-printed splints, cutting guides, and customized titanium plates for maxillary repositioning provides a highly accurate method for transferring virtual surgical planning to the operative field. Customized titanium plates demonstrated superior accuracy, particularly in anteroposterior movements, while offering clinical advantages such as ease of use, procedural efficiency, and a significant reduction in surgical time. These findings support the routine clinical integration of CAD/CAM technology in orthognathic surgery to enhance surgical precision and workflow.

This trial's limitations include a small, single-center sample size, which may restrict the generalizability of our results to various patient populations and surgical teams. While standardized protocols helped minimize variability, the individual experience of surgeons and the inherent learning curve associated with CAD/CAM workflows might have impacted accuracy. We did not assess long-term functional outcomes or patient-reported satisfaction, nor did we compare different virtual-planning software or 3D-printing platforms-elements that could influence transfer precision. Lastly, the customization parameters for our plates and splints may not be easily reproducible, which could hinder their widespread use.

## Conclusions

In this randomized clinical trial, both customized titanium plates and 3D-printed splints reliably translated virtual plans into the operative field for Le Fort I osteotomy. However, patient-specific titanium plates achieved superior precision (particularly in the anteroposterior dimension) while also reducing surgical time and eliminating intraoperative splint use. These results underscore the clinical value of CAD/CAM-driven, splintless workflows for enhancing accuracy and efficiency in orthognathic surgery.
